# Zoonotic infection with swine A/H1_av_N1 influenza virus in a child, Germany, June 2020

**DOI:** 10.2807/1560-7917.ES.2020.25.42.2001638

**Published:** 2020-10-22

**Authors:** Ralf Dürrwald, Marianne Wedde, Barbara Biere, Djin-Ye Oh, Martina Heßler-Klee, Christian Geidel, Renate Volmer, Anja Maria Hauri, Kai Gerst, Andrea Thürmer, Sandra Appelt, Janine Reiche, Susanne Duwe, Silke Buda, Thorsten Wolff, Walter Haas

**Affiliations:** 1 Robert Koch-Institut, Berlin, Germany; 2 Praxis in Hesse, Germany; 3 Landesbetrieb Hessisches Landeslabor (LHL), Fachgebiet II.4 Tiergesundheitsdienste, Gießen, Germany; 4 Gesundheitsamt in Hesse, Germany

**Keywords:** Germany, zoonotic infections, viral infections, influenza, influenza virus, sentinel surveillance, epidemiology, laboratory

## Abstract

A zoonotic A/sw/H1_av_N1 1C.2.2 influenza virus infection was detected in a German child that presented with influenza-like illness, including high fever. There was a history of close contact with pigs 3 days before symptom onset. The child recovered within 3 days. No other transmissions were observed. Serological investigations of the virus isolate revealed cross-reactions with ferret antisera against influenza A(H1N1)pdm09 virus, indicating a closer antigenic relationship with A(H1N1)pdm09 than with the former seasonal H1N1 viruses.

During routine surveillance at the National Influenza Centre in Germany in June 2020, a nasal swab was conspicuous because qPCR for the influenza A virus matrix protein (MP) and N1 neuraminidase (NA) genes were positive, whereas the haemagglutinin (HA) qPCR gave no results. The sample underwent whole genome sequencing and results pointed to a zoonotic influenza virus originating from swine. Here we describe the clinical features of the infection as well as the results of antigenic and genetic characterisation of this zoonotic influenza virus.

## Description of the case and setting

The diagnostic sample originated from a 2.5-year-old child who lived on a farm, had regular contact with pigs, most recently 3 days before symptom onset, and was not vaccinated against influenza. The child had influenza-like illness over 3 days, displaying fever up to 40 °C, a sore throat, rhinorrhoea, headaches, myalgias, some fussiness and one episode of emesis, and slept a lot. Afterwards, they recovered quickly and fully. The child was not treated with antiviral drugs. No other family member, including the child’s 5-month-old sibling, showed any symptoms, although some of them had been in close contact with the pigs. Four weeks later, 15 pigs of all age groups held at the farm and six family members were swabbed. All nasal swabs were negative, indicating absence of further virus circulation at this location. Four family members tested positive for rhinoviruses, but not the child who had had influenza. Because these swabs were qPCR-negative, virus isolation was not attempted from the pigs’ swabs.

The pig herd of the farm has 600 fattening pigs. Every 4 weeks, 120 new pigs (ca 30 kg, 8–9-weeks-old) are introduced from another farm in Germany. The pig farm is situated outside of the village and no one except the farmer, his family and the veterinarian have access to it. The pig feed is generated by the farm from its own harvest. The pigs are not vaccinated against influenza. Two weeks before the child was infected, a new batch of pigs arrived at the farm. At that time, some pigs were displaying a cough, for which they were treated with antibiotics. Thus, the infection was most probably introduced to the herd via the new batch of pigs. 

## Antigenic characterisation

Virus isolation from the child’s nasal swab was successful in MDCK-SIAT cells and embryonated hens’ eggs. The virus was termed influenza A/Hessen/47/2020 (HES/2020). Antigenic characterisation showed that cross-reactivity was highest with swine hyperimmune serum directed against influenza A/sw/H1_av_N1 virus (Table 1) [1]. Further investigations using ferret antisera demonstrated cross-reactivity with the wildtype and vaccine influenza A(H1N1)pdm09 viruses, but not with the previous seasonal influenza A(H1N1) viruses (i.e. those circulating before 2009). 

**Table 1:** Cross-reactivity of HES/2020 and other influenza A(H1N1) viruses investigated by haemagglutination inhibition using turkey erythrocytes, Germany, June 2020

Antiserum	Ferret antisera^a^					Swine hyperimmune sera^b^		
Virus	Brisbane/2/2018 A(H1N1)pdm09	Michigan/45/2015 A(H1N1)pdm09	California/7/2009 A(H1N1)pdm09	Brisbane/59/2007 seasonal H1N1	PR/8/1934 H1N1 34	2688/2010 A(H1N1)pdm09	12653/2010 A/sw/H1pdmN2	Re230/1992 A/sw/H1avN1
HES/2020^c^	1,280	1,280	640	< 10	< 10	160	< 10	2,560
Brisbane/2/2018 A(H1N1)pdm09	10,240	5,120	2,560	< 10	< 10	5,120	320	640
Michigan/45/2015 A(H1N1)pdm09	320	640	320	< 10	< 10	640	80	160
California/7/2009 A(H1N1)pdm09	80	160	320	< 10	< 10	1,280	160	160
Brisbane/59/2007 Seasonal H1N1	< 10	< 10	< 1:10	80	< 10	< 10	< 10	< 10
PR/8/1934 H1N1 of 1930s	< 10	< 10	< 1:10	< 10	1,280	160	80	80
Finistere/2899/1982 A/sw/H1avN1	320	40	80	< 10	< 10	80	< 10	640
Greven/2889/2004 /A/sw/H1avN1	< 10	< 10	< 10	< 10	< 10	< 10	< 10	320
Heinsberg/8905/2009 A/sw/H1avN1	10	< 10	< 10	< 10	< 10	< 10	< 10	320
2688/2010 A(H1N1)pdm09^d^	80	80	320	< 10	< 10	5,120	1,280	80
12653/2010 A/sw/H1pdmN2^d^	< 10	< 10	< 10	< 10	< 10	160	5,120	80
Re230 /1992 A/sw/H1avN1^d^	< 10	< 10	< 10	< 10	< 10	160	640	5,120

^a^ Post-infection sera of ferrets.

^b^ Hyperimmune sera of pigs were established according to [1].

^c^ Zoonotic influenza A/sw/H1_av_N1 virus (A/Hessen/47/2020) described in this study.

^d^ For genetic analysis of these viruses see also [18,29,30]; antisera against influenza A/sw/H1_pdm_N2 viruses cross-react minimally, or not at all, with A(H1N1)pdm09 and swine H1_av_N1 viruses because the antigenic distance is larger between them [29].

The Table shows reciprocal haemagglutination inhibition titres.

Blood samples from 14 of 15 pigs were found to be seropositive against the infecting virus (HES/2020). In haemagglutination inhibition (HI) tests against HES/2020, titres ranged from 1:10 to 1:160. All pig sera were negative against influenza A(H1N1)pdm09 virus (A/Brisbane/2/2018).

Sequence analysis showed that the majority of HA antigenic sites were conserved between influenza A/sw/H1_av_N1 and A(H1N1)pdm09 viruses (Table 2) [2]. In accordance with International Health Regulations, the case was reported to World Health Organization (WHO) via the Early Warning and Response System (EWRS) [3] and the virus was provided to the WHO Collaborating Centre London for further characterisation [4].

**Table 2:** Comparison of amino acids in the antigenic sites of the haemagglutinin molecule of HES/2020 vs influenza A(H1N1) viruses, Germany, June 2020

		Amino acid in the antigenic site^a^															
Site Sa									Site Sb						
Virus	HA clade/genotype	124	125	155	157	159	160	162	163	164	153	156	185	189	190	193	195
HES/2020	1C.2.2	P	N	G	S	P	K	R	N	S	K	N	D	Q	T	Q	N
swDUEL/2012	1C.2.2	P	N	G	S	P	K	R	K	S	K	N	D	Q	T	Q	N
swLUED/2013	1C.2.1	P	N	G	S	P	K	S	T	S	K	N	D	Q	T	Q	N
NL/2016	1C.2.1	P	N	E	S	P	K	S	T	S	K	N	D	Q	T	Q	N
swSHA/2013	1C.2.3/G1	P	N	G	S	P	K	S	K	S	K	N	D	Q	T	Q	N
swHEN/2018	1C.2.3/G4	P	N	G	S	P	K	S	K	S	K	N	D	Q	T	Q	N
swSHA/2014	1C.2.3/G5	P	N	G	S	P	K	S	K	S	K	N	D	Q	T	Q	N
swANH/2015	1C.2.3/G6	P	N	G	S	P	K	S	K	S	K	N	D	Q	T	Q	N
GU-MA/2019	pdm09	P	N	G	S	P	K	N	Q	T	K	N	I	E	S	Q	A
MICH/2015	pdm09	P	N	G	S	P	K	N	Q	S	K	N	T	Q	S	Q	A
		Site Ca1				Site Ca2						Site Cb					
Virus	HA clade/genotype	166	170	204	237	135	137	140	142	221	222	70	71	73	74	75	115
HES/2020	1C.2.2	T	G	S	G	A	S	G	N	R	E	L	L	A	N	S	E
swDUEL/2012	1C.2.2	T	G	S	G	A	S	G	N	R	E	L	L	A	N	S	E
swLUED/2013	1C.2.1	T	G	S	G	A	S	G	K	R	E	L	I	A	N	S	E
NL/2016	1C.2.1	T	G	S	G	A	S	G	K	R	E	L	I	A	N	S	E
swSHA/2013	1C.2.3/G1	T	G	S	G	A	S	G	N	R	G	L	L	A	N	S	E
swHEN/2018	1C.2.3/G4	T	G	T	G	S	S	G	N	R	E	L	L	A	N	S	E
swSHA/2014	1C.2.3/G5	T	G	S	G	S	S	G	N	R	E	L	L	A	N	S	E
swANH/2015	1C.2.3/G6	T	G	S	G	A	S	G	N	R	E	L	L	A	N	S	E
GU-MA/2019	pdm09	I	G	S	G	A	P	G	K	R	D	L	S	A	R	S	E
MICH/2015	pdm09	I	G	S	G	A	P	G	K	R	D	L	S	A	S	S	E

HA: haemagglutinin.

^a^ H1 numbering without signal sequence.

Virus names from top to bottom: A/Hessen/47/2020, A/swine/Duelmen/15075/2012, A/swine/Luedinghausen/18391/2013, A/Netherlands/3315/2016, A/swine/Shandong/39/2013, A/swine/Henan/SN13/2018, A/swine/Shandong/S113/2014, A/swine/Anhui/1227/2015, A/Guangdong-Maonan/SWL1536/2019, A/Michigan/45/2015.

Shaded cells: amino acid differences relative to HES/2020; presentation of antigenic sites adapted from [2].

## Genetic characterisation

The genetic classification of HES/2020 is F (polymerase basic protein 2, PB2), G (polymerase basic protein 1, PB1), I (polymerase acidic protein, PA), 1C.2.2 (HA), F (nucleoprotein, NP), 1F (NA), F (MP), 1E (nonstructural proteins, NS) [5,6]. It is unrelated to the recently reported G4 reassortant EA(H1N1) viruses circulating in China [2]. Sequences were submitted to GISAID and the accession numbers were as follows: PB2: EPI1757436, PB1: EPI1757437, PA: EPI1757435, HA: EPI1757439, NP: EPI1757432, NA: EPI1757438, MP: EPI1757434 and NS: EPI1757433. Blast analysis and phylogenetic analysis demonstrated that the segments of HES/2020 are closely related to those of different viruses: HA (Figure) and NA to influenza A/swine/Germany/Ellerbrock-IDT14696/2012 (swELLE/2012, H1N1, HA-1C.2.2) and A/swine/Duelmen/15075/2012 (swDUEL/2012, H1N1, HA-1C.2.2); MP, NP, NS and PB1 to A/swine/Luedinghausen/18391/2013 (swLUED/2013, H1N1, HA-1C.2.1) and to zoonotic A/Netherlands/3315/2016 (NL/2016, H1N1, HA-1C.2.1) [7]; PA and PA-X to A/swine/Belgium/Heist-op-den-Berg-363/2012 (swHEIST/2012, H1N1, HA-1C.2.1); and PB2 to A/swine/Belgium/Oostkamp-26/2012 (swOOST/2012, H1N2, HA-1B.1.2.1). The genetic composition of HES/2020 indicates several intra- and inter-clade reassortments.

**Figure:**
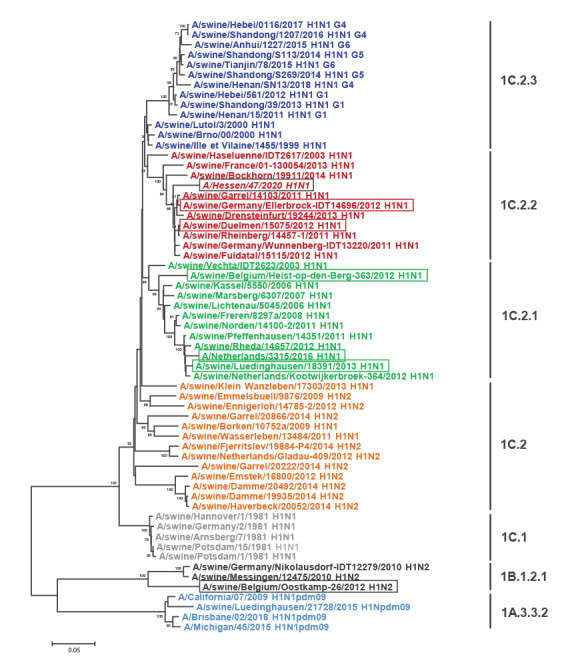
Phylogenetic analysis of the haemagglutinin gene (1,695 bp) of influenza A viruses

The phylogenetic analyses of the other coding sequences (NA, MP, NP, NS, NS1, PA, PA-X, PB1, PB1-F2, PB2) are shown in Supplementary Figures S1–S10. Virus genomes were analysed by whole genome sequencing and were phylogenetically evaluated with Mega7 (neighbour-joining method, midpoint rooted, bootstrap test with 1,000 replicates, Kimura 2-parameter method, partial deletion (site coverage cut-off: 5%). Sixty-one influenza A viruses were characterised: 1A.3.3.2/H1N1pdm09 (light blue), 1B.1.2.1 (black), 1C.1 (grey), 1C.2 including reassorted A(H1N2)-viruses (orange), 1C.2.1 (green), 1C.2.2 including zoonotic A/Hessen/47/2020 (red, italics, framed in black) and 1C.2.3 including genotypes G1/G4/G5/G6 (blue) [2]. Framed items: closely related viruses that are identified by BLAST analysis of each segment (data not shown) and used as reference viruses for further analysis: swELLE/2012 and swDUEL/2012 for HA and NA, zoonotic NL/2016 and swLUED/2013 for MP, NP, NS and PB1, swHEIST/2012 for PA and swOOST/2012 for PB2.

Nucleotide sequence variation was highest over the usually well conserved NP and PA-X coding sequences (Twelve coding sequences were analysed: HA, NA, M1, M2, NP, NS1, NEP, PA, PA-X, PB1, PB1-F2, PB2 with a length of 1,701, 1,410, 759, 294, 1,497, 693, 366, 2,151, 759, 2,274, 273, 2,280 nt, respectively). They displayed nucleotide identities of 95% each, whereas all other coding sequences displayed nucleotide sequence identity > 95% relative to the reference sequence. Reference sequences were swDUEL/2012 for HA and NA, swLUED/2013 for MP, NP, NS and PB1, swHEIST/2012 for PA and swOOST/2012 for PB2). Amino acid (AA) sequence variation was highest over the regulator proteins of the host innate immune response, NS1, PA-X and PB1-F2 (identities of 95%, 94% and 95%, respectively) [8,9]. Variant calling for HES/2020 and another zoonotic virus, NL/2016 [7], relative to the reference viruses, demonstrated that the number of substitutions common to both HES/2020 and another zoonotic virus, NL/2016, was highest for the PB1-F2 protein (four of five substitutions) Table 3). In contrast to NL/2016, PB1-F2 of HES/2020 is full-length at 90 AA. Phylogenetic analyses of MP, NP, NS, NS1, PB1 and PB1-F2 demonstrated that the two zoonotic viruses are closely related (Figure, Supplementary Figures S1–S10). To detect substitutions with potential functional relevance in the HES/2020 genome, the FluSurver online tool was employed (https://flusurver.bii.a-star.edu.sg/), identifying substitutions in the HA receptor binding domain (D222E) [10], NP (K48Q;R98K;R99K [11], R351K;V353I;Q357K [12]) and PB2 (D701N) [13] (Supplementary Table S1). The substitutions NP-Q357K, PA-X-R57K, PA-R57K, PA-T639A are present in both zoonotic viruses and in both analyses (FluSurver and the genetic comparison in Table 3).

**Table 3:** Non-synonymous substitutions in the coding sequences of HES/2020 relative to closely related swine influenza viruses and common substitutions with the zoonotic NL/2016 virus, Germany, June 2020

CDS	Substitutions of HES/2020 relative to reference sequences^a^															
**HA1^b^**	**T14A^c^**	**G53K**	**V57L**	**I80V**	***K163N***	**I214T**	**M227I**	**H253Y**	**V265I**	**T267M**	**D269N**	***H271R***	**K278M**	**K302E**	**Q311H**	
HA2^b^	**Q353H**	Q365R	***D399G***	S451A	N473D	**D474E**										
NA	M15L	A76V	***A79E***	S82P	L140M	D210S	**V211I**	K220R	A232V	E311D	V338I	***T340I***	S369N	V389I	***T396I***	**N398D**
M1	G30S															
M2	**T28I**	F48S														
NP	**S16G**	***K105V***	**Q357K**	V363I	A423T	R452K	S482N	N498S								
NS1	**K44R**	S48N	R67C	A86T	R88H	M98I	V111L	I123V	**Y165S**	A191T	N209I	T215I				
NEP	K18R	T52S	L55H													
PA	H24Y	**R57K**	***I66S***	**R104K**	I184L	**K204R**	E206D	E252G	**I268L**	**K269R**	L335I	***H346N***	M374V	***G388S***	**T639A**	**V712M**
PA-X	H24Y	**R57K**	***I66S***	**R104K**	I184L	R199K	**N204D**	K206T	S207L	E209G	**T212I**	**I216T**	S219F	P224L	K252E	
PB1	I69V	I111M	**K213N**	**K571R**	V632I	G636E	V640I	A648S	I682V	**S741A**						
PB1-F2	**T39M**	**S63F**	**K73R**	*stop80W*	**K81R**											
PB2	S12L	I255V	A351T	K353R	R389K	C409R	M473V	**A598T**	D611E							

AA: amino acid; CDS: coding sequences; HES/2020: influenza A/sw/H1avN1 (A/Hessen/47/2020).

^a^ Reference sequences were as follows: swDUEL/2012 for HA and NA, swLUED/2013 for MP, NP, NS and PB1, swHEIST/2012 for PA and swOOST/2012 for PB2.

^b^ H1 numbering without signal sequence.

^c^ AA substitution within the HA signal sequence.

Consistent AA substitutions that occur in both zoonotic viruses HES/2020 and NL/2016 virus are labelled in bold; AA substitutions that differ from the reference viruses and between zoonotic HES/2020 and zoonotic NL/2016 virus are labelled in bold and italics and only the AA of HES/2020 is displayed; the change tag to tgg at codon 80 revealed an extension of HES/2020 PB1-F2 to 90 AA and is shown in italics.

## Resistance characterisation

While HES/2020 does not exhibit NA or PA mutations conferring resistance against neuraminidase inhibitors or baloxavir marboxil, its M2 sequence contains the AA substitutions L26I, V27A and S31N, all of which are associated with adamantane resistance (amantadine and rimantadine). Phenotypic susceptibility testing against oseltamivir, peramivir and zanamivir confirmed that HES/2020 was sensitive to all neuraminidase inhibitors authorised in Europe.

## Discussion

This is the sixth zoonotic swine influenza virus infection in humans investigated at the German National Influenza Centre (in 2007: A/sw/H1_av_N1 and A/sw/H3N2 in Lower Saxony, in 2010: A/sw/H1_av_N1 in Lower Saxony, in 2011: A/sw/H1_hu_N2 and A/sw/H1_av_N1 in Lower Saxony) [14]. Of the five previously reported cases, two occurred in children and one in an immunocompromised adult; influenza A/sw/H1_av_N1 infections were the most common [14]. All previous German cases were detected in Lower Saxony, the federal state with the second largest pig population in Germany. The case described here is the first from a region with a low density of pig holdings, i.e. Hesse. 

The genetic diversity of influenza A viruses in the European pig population is increasing [15-17]. A/sw/H1_av_N1 are the predominant swine influenza viruses in Germany [18]. Among them, the two most prevalent lineages are H1_av_N1 1C.2.2 and H1_av_N1 1C.2.1. Other swine influenza viruses include H1_hu_N2 and H3N2 viruses as well as H1_pdm_N1 and H1_pdm_N2 viruses [15-18]. An increasing number of reassortments between these viruses augment the diversity of influenza virus populations in swine.

Swine influenza viruses acquired adamantane resistance in the late 1980s [19]. The influenza A(H1N1)pdm09 virus contains the MP gene from A/sw/H1_av_N1 viruses which confers adamantane resistance via the M2-S31N mutation in MP gene 2 [20]. This mutation was common in all seasonal influenza A viruses circulating globally during the last years [21]. In addition to S31N, HES/2020 contains the M2 AA substitutions L26I, V27A which are also associated with adamantane resistance. The M2-L26I and M2-V27A mutations can be found sporadically in influenza A viruses [21]. 

Swine influenza viruses have acquired some resistance genes against human myxovirus resistance protein MxA during their evolution in pigs, facilitating their transmission to humans [12]. Pig-to-human influenza virus transmissions are not rare, especially in close contact settings such as agricultural fairs [22], and sporadic zoonotic transmission of swine influenza A(H1N1) virus has been reported [23,24]. The farm child was the only member of his family who was infected, although some of the other family members had also been exposed. The infection of a child is not surprising. Because of their limited exposure history, young children display a narrower (if any) immune response to influenza virus than adults [25].

Our serology investigations indicate some level of cross-reactivity between influenza A(H1N1)pdm09 virus and A/sw/H1_av_N1 viruses in ferrets. This is in line with previous findings that influenza A(H1N1)pdm09 infection induces broadly neutralising (not strain-specific) antibodies [26]. Antibodies against influenza A/sw/H1_av_N1 viruses in the human population are rare [27,28]. On the other hand, sera of human volunteers collected 3–7 weeks after vaccination with the annual 2017/18 vaccine all reflected antibodies against influenza A/sw/H1_av_N1 virus at varying microneutralisation titres and none was negative [15]. Although the family members of the zoonotic case had not been vaccinated, they may have been exposed to human and swine influenza A viruses before, potentially resulting in pre-existing immunity which might impair transmission of influenza A/sw/H1_av_N1 influenza virus. 

However, the rising genetic diversity among swine influenza viruses, involving antigenic drift and shift, may increase divergence from influenza A/sw/H1_av_N1 viruses in the future. In particular, swine reassortant viruses may quickly acquire antigenic changes, and this is where substantial zoonotic potential may arise.

## Supplementary Data

74600 Supplement
