# Supplementary material for: Zoonotic infection with swine A/H1avN1 influenza virus in a child, Germany, June 2020
Source: Euro Surveill. 2020 Oct 22;25(42):2001638. doi: 10.2807/1560-7917.ES.2020.25.42.2001638 (PMC7651875; doi:10.2807/1560-7917.ES.2020.25.42.2001638)
Supplement: Supplement [file 20-01638_DURRWALD_Supplement.pdf]

Supplementary information for

Zoonotic infection with swine A/H1$_{av}$N1 influenza virus in a German child

This PDF file includes:

Figures S1 to S10

Table S1

The supplementary material is hosted by *Eurosurveillance* as supporting information alongside the article Zoonotic infection with swine A/H1$_{av}$N1 influenza virus in a German child, on behalf of the authors, who remain responsible for the accuracy and appropriateness of the content. The same standards for ethics, copyright, attributions and permissions as for the article apply. Supplements are not edited by *Eurosurveillance* and the journal is not responsible for the maintenance of any links or email addresses provided therein.

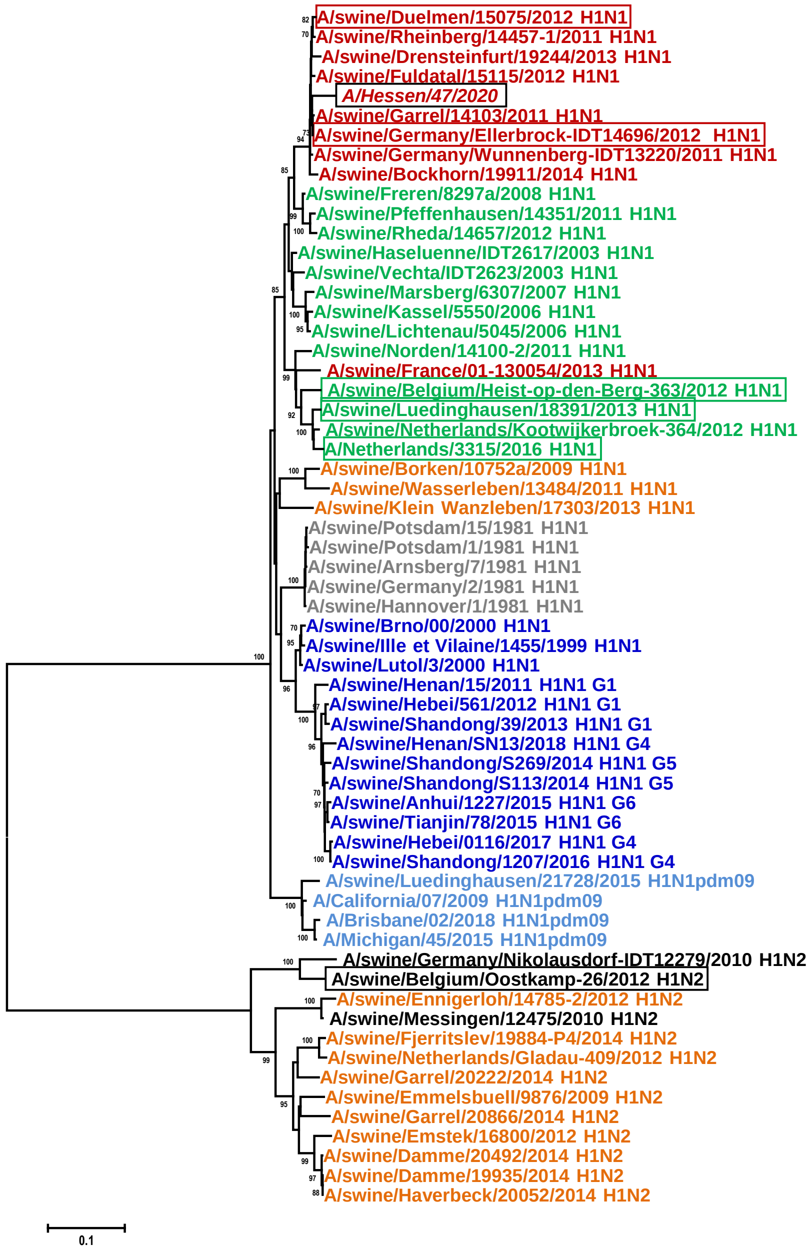

Figure S1: Phylogenetic analysis of NA gene of influenza A viruses. Virus genomes were analyzed by NGS and were phylogenetically evaluated with Mega7 (Neighbor-Joining method, midpoint rooted, bootstrap test with 1000 replicates, Kimura 2-parameter method, partial deletion (site coverage cutoff 5%). Sixty-one influenza A viruses were characterized: 1A.3.3.2/H1N1pdm09 (light blue), 1B.1.2.1 (black), 1C.1 (grey), 1C.2 including reassorted A(H1N2)-viruses (orange), 1C.2.1 (green), 1C.2.2 including zoonotic A/Hessen/47/2020 (red, italics, framed in black) and 1C.2.3 including genotypes G1/G4/G5/G6 (blue, Sun 2020 [3]). Closely related viruses that are identified by Blast analysis of each segment (data not shown) and used as reference viruses for further analysis, respectively, are framed: For HA and NA, swELLE/2012 and swDUEL/2012; for MP, NP, NS, NS1, PB1 and PB1-F2, zoonotic NL/2016 and swLUED/2013; for PA and PA-X, swHEIST/2012; and for PB2, swOOST/2012

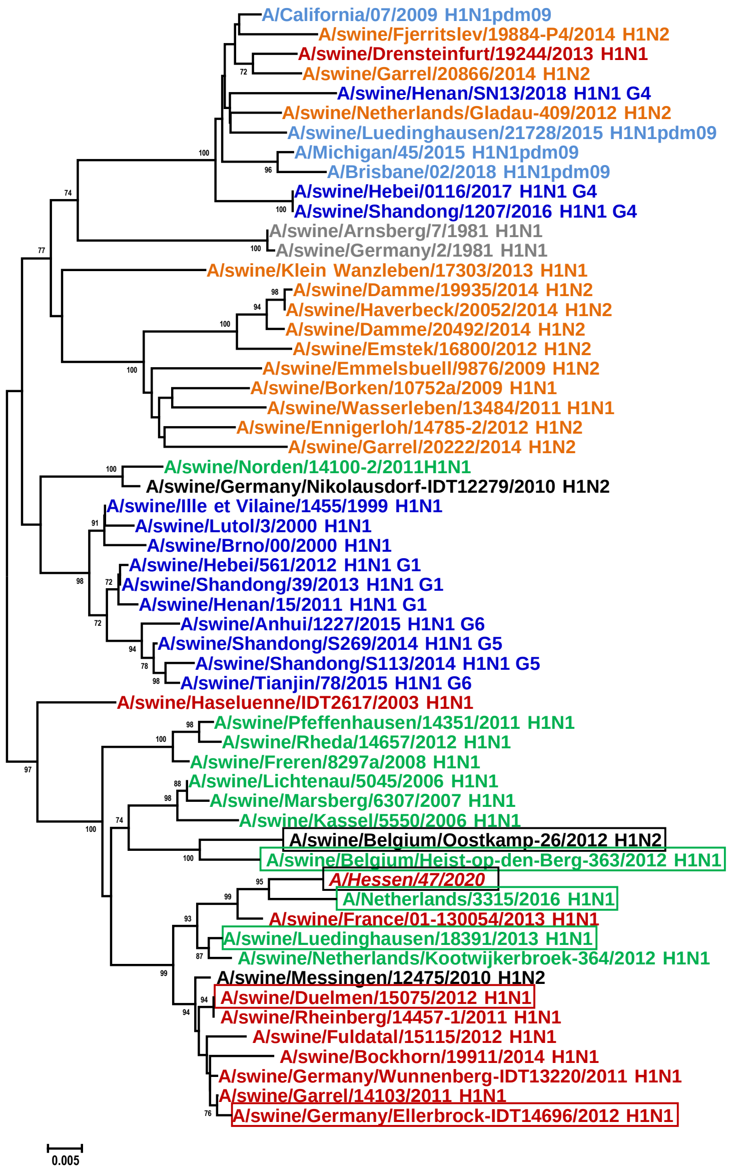

Figure S2: Phylogenetic analysis of MP gene of influenza A viruses. Virus genomes were analyzed by NGS and were phylogenetically evaluated with Mega7 (Neighbor-Joining method, midpoint rooted, bootstrap test with 1000 replicates, Kimura 2-parameter method, partial deletion (site coverage cutoff 5%). Sixty-one influenza A viruses were characterized: 1A.3.3.2/H1N1pdm09 (light blue), 1B.1.2.1 (black), 1C.1 (grey), 1C.2 including reassorted A(H1N2)-viruses (orange), 1C.2.1 (green), 1C.2.2 including zoonotic A/Hessen/47/2020 (red, italics, framed in black) and 1C.2.3 including genotypes G1/G4/G5/G6 (blue, Sun 2020 [3]). Closely related viruses that are identified by Blast analysis of each segment (data not shown) and used as reference viruses for further analysis, respectively, are framed: For HA and NA, swELLE/2012 and swDUEL/2012; for MP, NP, NS, NS1, PB1 and PB1-F2, zoonotic NL/2016 and swLUED/2013; for PA and PA-X, swHEIST/2012; and for PB2, swOOST/2012

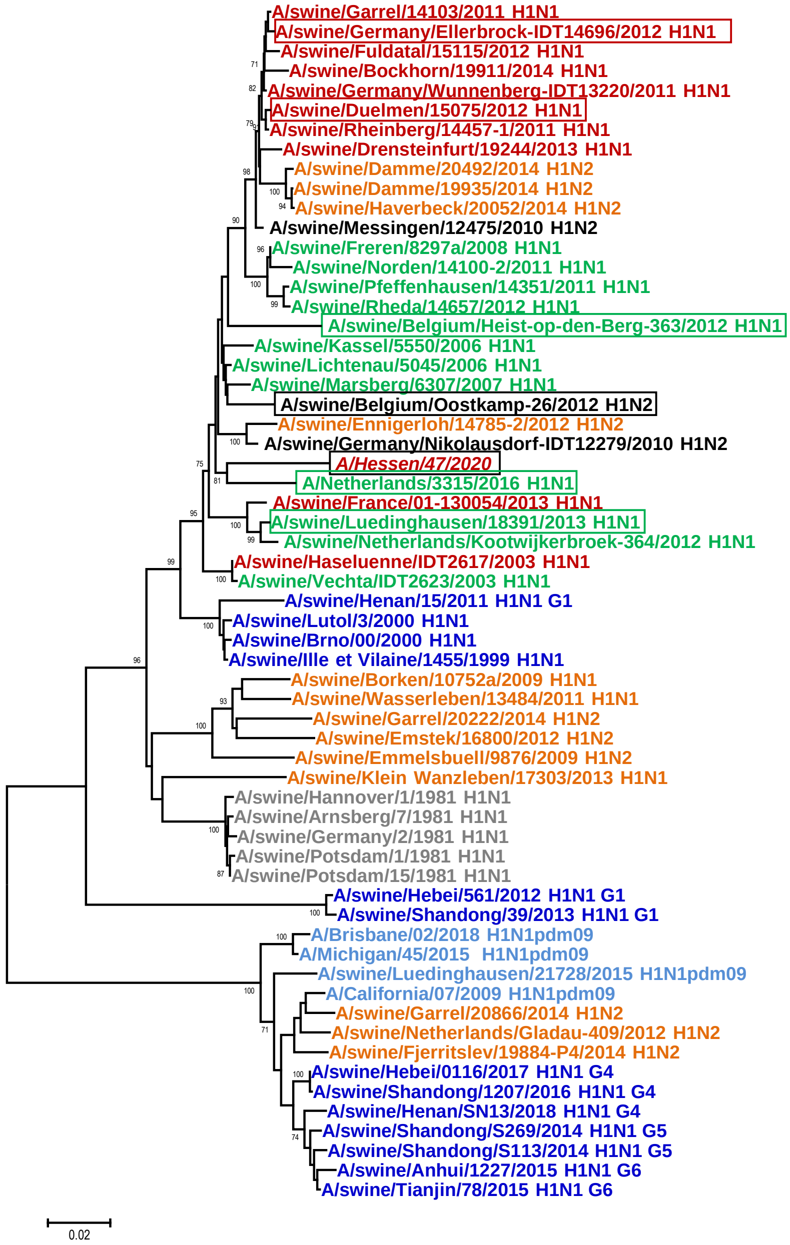

Figure S3: Phylogenetic analysis of NP gene of influenza A viruses. Virus genomes were analyzed by NGS and were phylogenetically evaluated with Mega7 (Neighbor-Joining method, midpoint rooted, bootstrap test with 1000 replicates, Kimura 2-parameter method, partial deletion (site coverage cutoff 5%). Sixty-one influenza A viruses were characterized: 1A.3.3.2/H1N1pdm09 (light blue), 1B.1.2.1 (black), 1C.1 (grey), 1C.2 including reassorted A(H1N2)-viruses (orange), 1C.2.1 (green), 1C.2.2 including zoonotic A/Hessen/47/2020 (red, italics, framed in black) and 1C.2.3 including genotypes G1/G4/G5/G6 (blue, Sun 2020 [3]). Closely related viruses that are identified by Blast analysis of each segment (data not shown) and used as reference viruses for further analysis, respectively, are framed: For HA and NA, swELLE/2012 and swDUEL/2012; for MP, NP, NS, NS-1, PB1 and PB1-F2, zoonotic NL/2016 and swLUED/2013; for PA and PA-X, swHEIST/2012; and for PB2, swOOST/2012

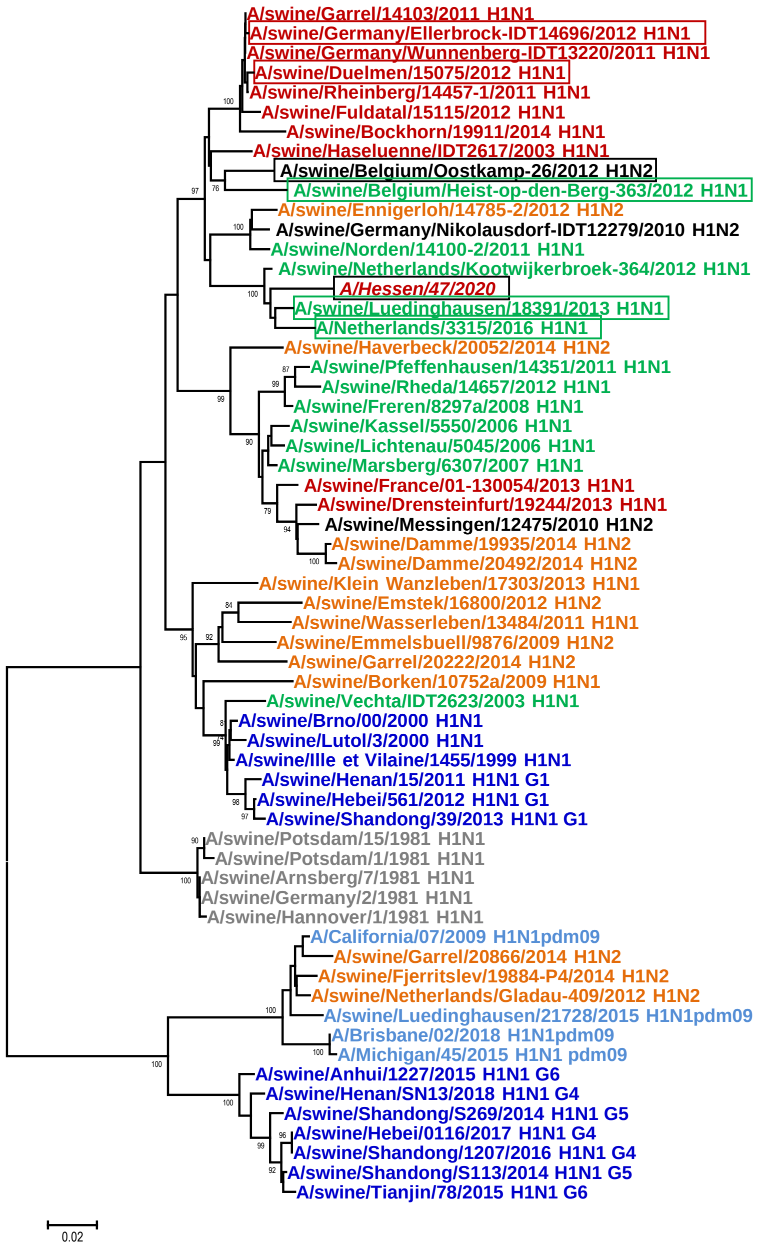

Figure S4: Phylogenetic analysis of NS gene of influenza A viruses. Virus genomes were analyzed by NGS and were phylogenetically evaluated with Mega7 (Neighbor-Joining method, midpoint rooted, bootstrap test with 1000 replicates, Kimura 2-parameter method, partial deletion (site coverage cutoff 5%). Sixty-one influenza A viruses were characterized: 1A.3.3.2/H1N1pdm09 (light blue), 1B.1.2.1 (black), 1C.1 (grey), 1C.2 including reassorted A(H1N2)-viruses (orange), 1C.2.1 (green), 1C.2.2 including zoonotic A/Hessen/47/2020 (red, italics, framed in black) and 1C.2.3 including genotypes G1/G4/G5/G6 (blue, Sun 2020 [3]). Closely related viruses that are identified by Blast analysis of each segment (data not shown) and used as reference viruses for further analysis, respectively, are framed: For HA, NA, swELLE/2012 + swDUEL/2012; for MP, NP, NS, NS1, PB1, PB1-F2, zoonotic NL/2016 and swLUED/2013; for PA, PA-X, swHEIST/2012; and for PB2, swOOST/2012

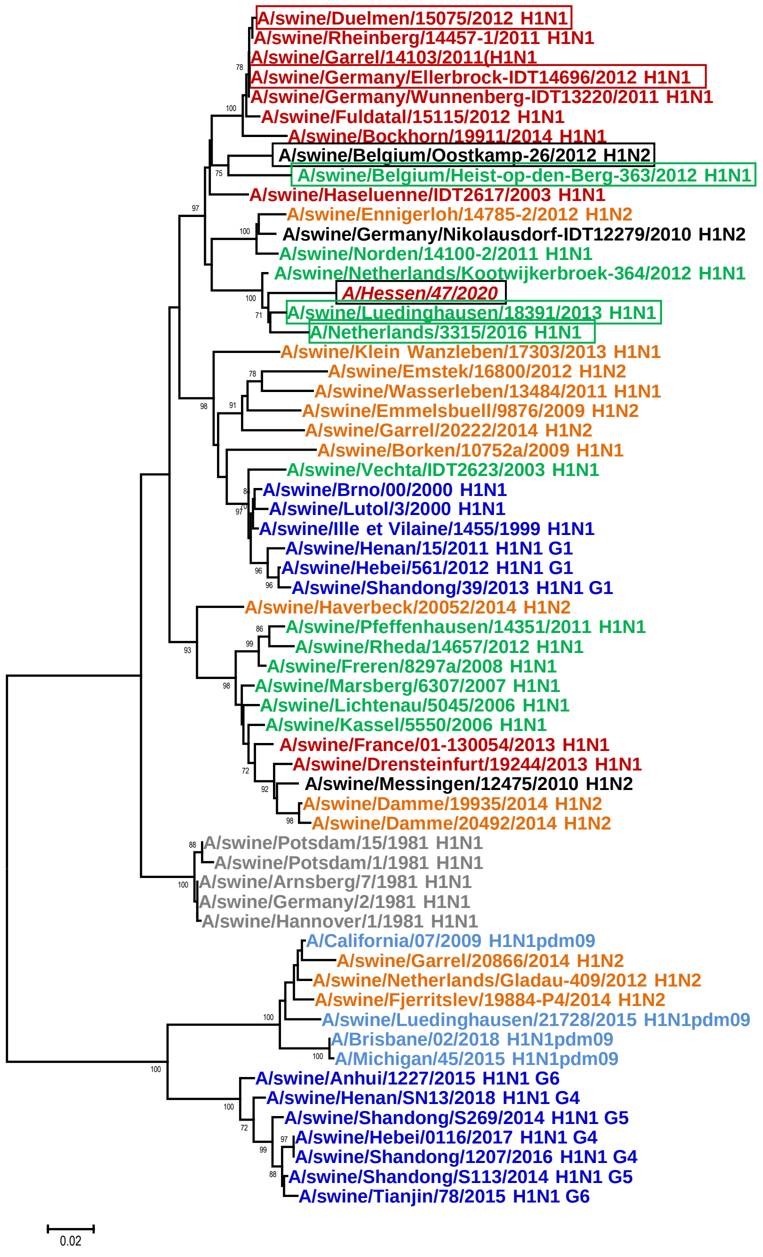

Figure S5: Phylogenetic analysis of NS1 gene of influenza A viruses. Virus genomes were analyzed by NGS and were phylogenetically evaluated with Mega7 (Neighbor-Joining method, midpoint rooted, bootstrap test with 1000 replicates, Kimura 2-parameter method, partial deletion (site coverage cutoff 5%). Sixty-one influenza A viruses were characterized: 1A.3.3.2/H1N1pdm09 (light blue), 1B.1.2.1 (black), 1C.1 (grey), 1C.2 including reassorted A(H1N2)-viruses (orange), 1C.2.1 (green), 1C.2.2 including zoonotic A/Hessen/47/2020 (red, italics, framed in black) and 1C.2.3 including genotypes G1/G4/G5/G6 (blue, Sun 2020 [3]). Closely related viruses that are identified by Blast analysis of each segment (data not shown) and used as reference viruses for further analysis, respectively, are framed: For HA, NA, swELLE/2012 and swDUEL/2012; for MP, NP, NS, NS1, PB1, PB1-F2, zoonotic NL/2016 + swLUED/2013; for PA, PA-X, swHEIST/2012; and for PB2, swOOST/2012

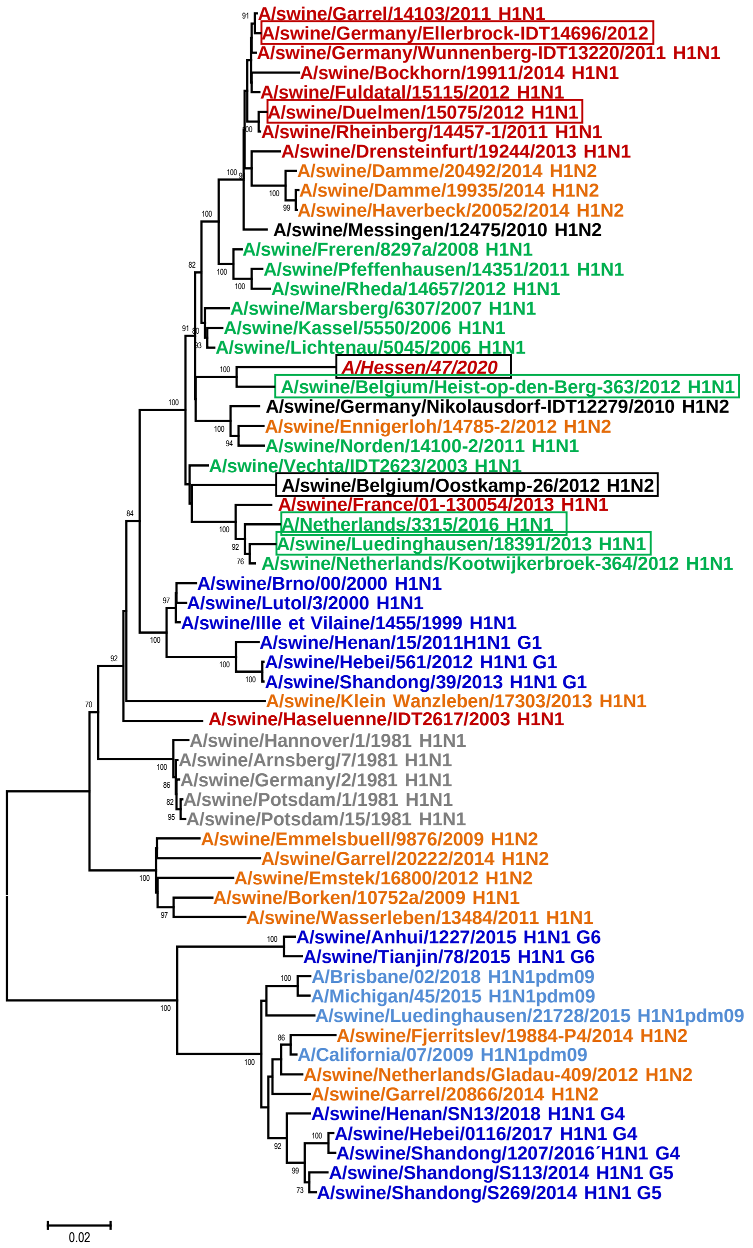

Figure S6: Phylogenetic analysis of PA gene of influenza A viruses. Virus genomes were analyzed by NGS and were phylogenetically evaluated with Mega7 (Neighbor-Joining method, midpoint rooted, bootstrap test with 1000 replicates, Kimura 2-parameter method, partial deletion (site coverage cutoff 5%). Sixty-one influenza A viruses were characterized: 1A.3.3.2/H1N1pdm09 (light blue), 1B.1.2.1 (black), 1C.1 (grey), 1C.2 including reassorted A(H1N2)-viruses (orange), 1C.2.1 (green), 1C.2.2 including zoonotic A/Hessen/47/2020 (red, italics, framed in black) and 1C.2.3 including genotypes G1/G4/G5/G6 (blue, Sun 2020 [3]). Closely related viruses that are identified by Blast analysis of each segment (data not shown) and used as reference viruses for further analysis, respectively, are framed: For HA, NA, swELLE/2012 and swDUEL/2012; for MP, NP, NS, NS1, PB1, PB1-F2, zoonotic NL/2016 + swLUED/2013; for PA, PA-X, swHEIST/2012; and for PB2, swOOST/2012

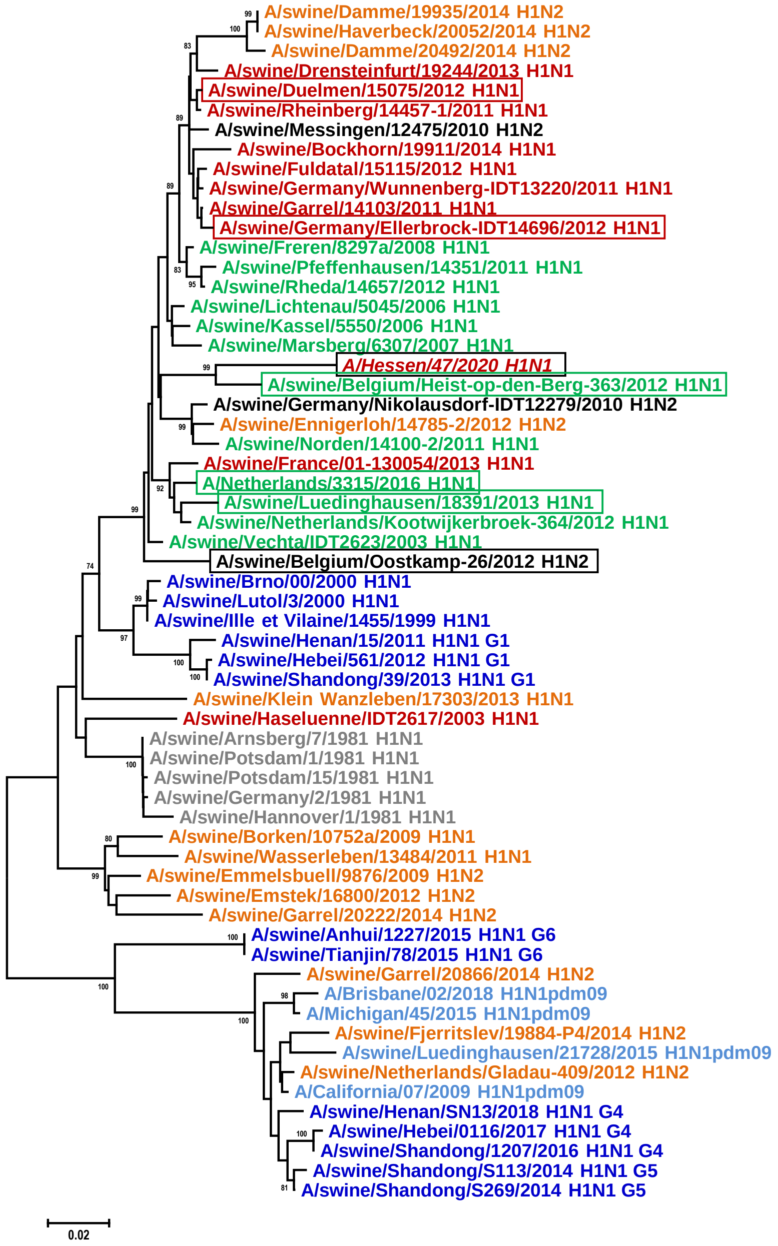

Figure S7: Phylogenetic analysis of PA-X gene of influenza A viruses. Virus genomes were analyzed by NGS and were phylogenetically evaluated with Mega7 (Neighbor-Joining method, midpoint rooted, bootstrap test with 1000 replicates, Kimura 2-parameter method, partial deletion (site coverage cutoff 5%). Sixty-one influenza A viruses were characterized: 1A.3.3.2/H1N1pdm09 (light blue), 1B.1.2.1 (black), 1C.1 (grey), 1C.2 including reassorted A(H1N2)-viruses (orange), 1C.2.1 (green), 1C.2.2 including zoonotic A/Hessen/47/2020 (red, italics, framed in black) and 1C.2.3 including genotypes G1/G4/G5/G6 (blue, Sun 2020 [3]). Closely related viruses that are identified by Blast analysis of each segment (data not shown) and used as reference viruses for further analysis, respectively, are framed: For HA, NA, swELLE/2012 and swDUEL/2012; for MP, NP, NS, NS1, PB1, PB1-F2, zoonotic NL/2016 and swLUED/2013; for PA, PA-X swHEIST/2012; and for PB2, swOOST/2012

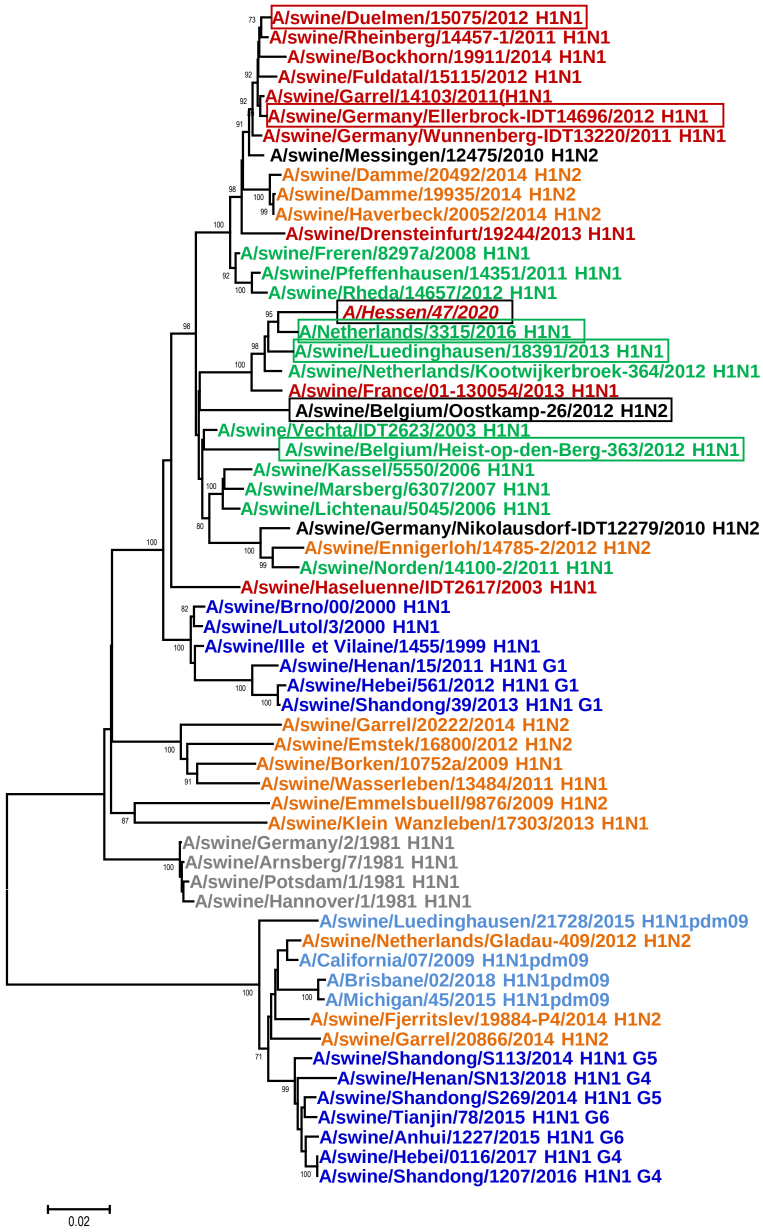

Figure S8: Phylogenetic analysis of PB1 gene of influenza A viruses. Virus genomes were analyzed by NGS and were phylogenetically evaluated with Mega7 (Neighbor-Joining method, midpoint rooted, bootstrap test with 1000 replicates, Kimura 2-parameter method, partial deletion (site coverage cutoff 5%). Sixty-one influenza A viruses were characterized: 1A.3.3.2/H1N1pdm09 (light blue), 1B.1.2.1 (black), 1C.1 (grey), 1C.2 including reassorted A(H1N2)-viruses (orange), 1C.2.1 (green), 1C.2.2 including zoonotic A/Hessen/47/2020 (red, italics, framed in black) and 1C.2.3 including genotypes G1/G4/G5/G6 (blue, Sun 2020 [3]). Closely related viruses that are identified by Blast analysis of each segment (data not shown) and used as reference viruses for further analysis, respectively, are framed: For HA, NA, swELLE/2012 and swDUEL/2012; for MP, NP, NS, NS1, PB1, PB1-F2, zoonotic NL/2016 + swLUED/2013; for PA, PA-X swHEIST/2012; and for PB2, swOOST/2012

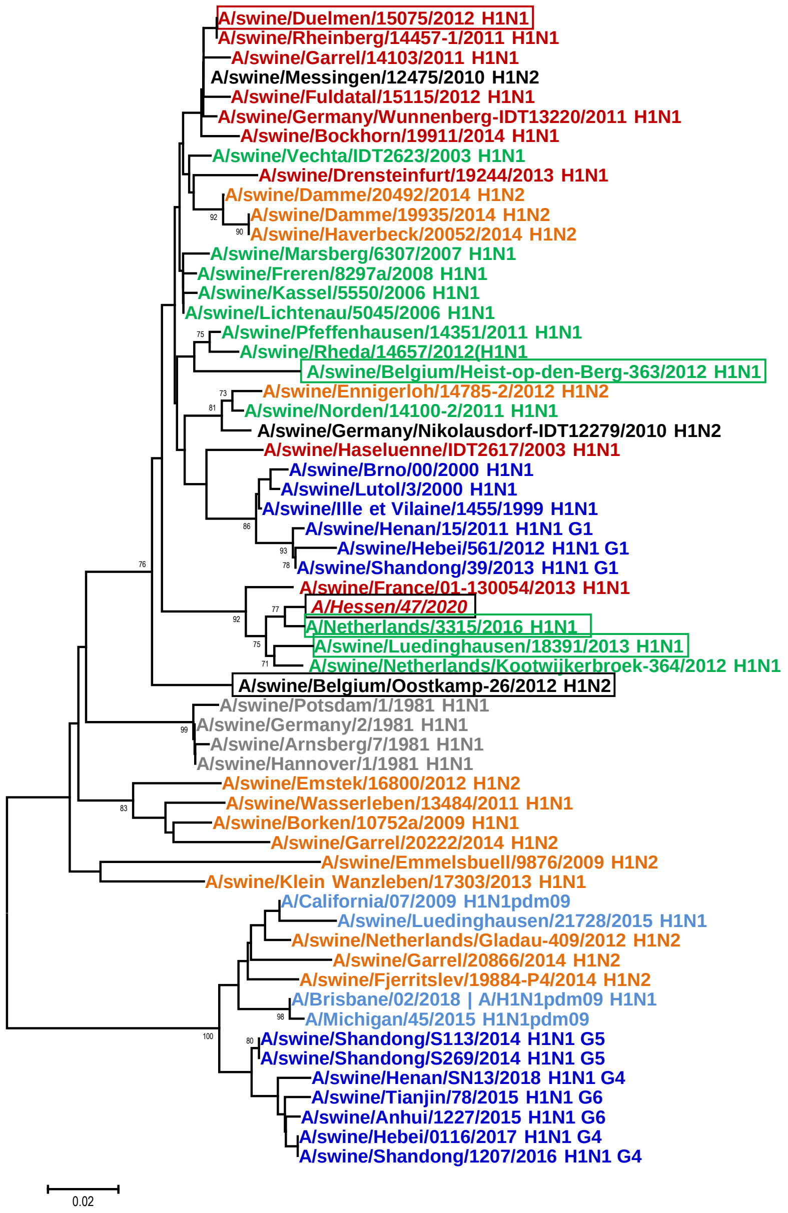

Figure S9: Phylogenetic analysis of PB1-F2 gene of influenza A viruses. Virus genomes were analyzed by NGS and were phylogenetically evaluated with Mega7 (Neighbor-Joining method, midpoint rooted, bootstrap test with 1000 replicates, Kimura 2-parameter method, partial deletion (site coverage cutoff 5%). Sixty-one influenza A viruses were characterized: 1A.3.3.2/H1N1pdm09 (light blue), 1B.1.2.1 (black), 1C.1 (grey), 1C.2 including reassorted A(H1N2)-viruses (orange), 1C.2.1 (green), 1C.2.2 including zoonotic A/Hessen/47/2020 (red, italics, framed in black) and 1C.2.3 including genotypes G1/G4/G5/G6 (blue, Sun 2020 [3]). Closely related viruses that are identified by Blast analysis of each segment (data not shown) and used as reference viruses for further analysis, respectively, are framed: For HA, NA, swELLE/2012 and swDUEL/2012; for MP, NP, NS, NS1, PB1, PB1-F2, zoonotic NL/2016 and swLUED/2013; for PA, PA-X swHEIST/2012; and for PB2, swOOST/2012

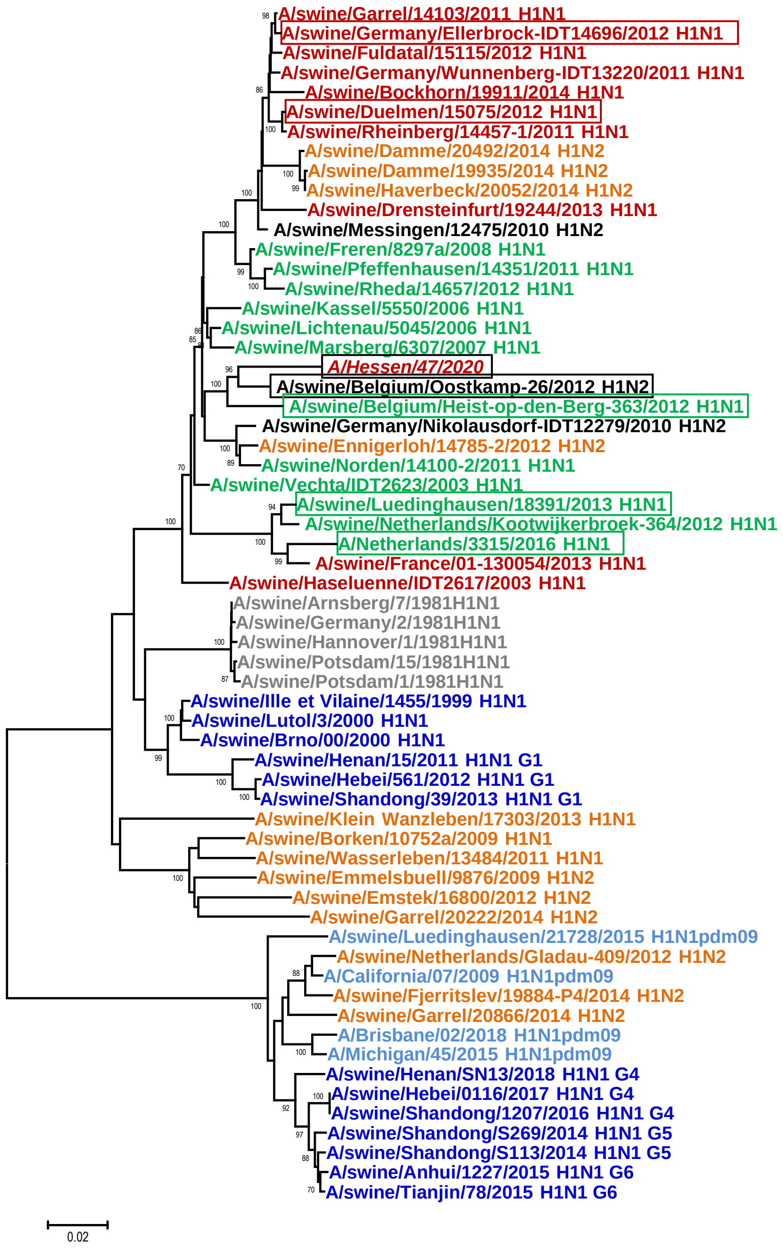

Figure S10: Phylogenetic analysis of PB2 gene of influenza A viruses. Virus genomes were analyzed by NGS and were phylogenetically evaluated with Mega7 (Neighbor-Joining method, midpoint rooted, bootstrap test with 1000 replicates, Kimura 2-parameter method, partial deletion (site coverage cutoff 5%). Sixty-one influenza A viruses were characterized: 1A.3.3.2/H1N1pdm09 (light blue), 1B.1.2.1 (black), 1C.1 (grey), 1C.2 including reassorted A(H1N2)-viruses (orange), 1C.2.1 (green), 1C.2.2 including zoonotic A/Hessen/47/2020 (red, italics, framed in black) and 1C.2.3 including genotypes G1/G4/G5/G6 (blue, Sun 2020 [3]). Closely related viruses that are identified by Blast analysis of each segment (data not shown) and used as reference viruses for further analysis, respectively, are framed: For HA, NA, swELLE/2012 and swDUEL/2012; for MP, NP, NS, NS-1, PB1, PB1-F2, zoonotic NL/2016 and swLUED/2013; for PA, PA-X swHEIST/2012; and for PB2, swOOST/2012

Table S1: Results of FluServer analysis ((https://flusurver.bii.a-star.edu.sg/)

Query	All Mutations	Red Mutations	Orange Mutations	Reference
HA	A3V;R4K;L6F;L9F;A11S;A13T;T15L;N16K;I22V;V36I;D52T;K60S;K62N;I64K;G70K;K71N;I74L;L78I;S91N;S100P;N101D;E103K;D111E;I113A;S124T;K130R;T137A;S138T;E144D;K147R;V149T;A151I;Y155H;A156S;S159N;L168I;T169V;S173N;S179R;K180N;V183T;L193I;G202D;T203S;S207T;A212N;D213H;A214T;V216I;N224Y;R225Q;I231T;A232V;V237I;D239E;M244I;E252D;P253Q;R276K;G279S;T284M;D286N;P288R;D291N;N293T;K295M;I303L;N304K;S306N;I312V;V315I;R325K;K328H;Q382R;N387I;T393S;V399I;T408A;A409S;N416G;R419K;I435V;V444I;S457F;R460K;S468A;K471R;A491E;R497K;D502N;E516K;Y528H	S173N	L9F;S100P;D111E;K147R;A151I;A156S;S159N;T169V;A212N;N224Y;A232V;D239E;P253Q;D291N;N304K;S306N	HA A/BrevigMission/1/1918(H1N1)
MP	M248I			M1 A/California/07/2009(H1N1)
MP	T11I;C19Y;N20S;P25A;L26I;V27A;S31N;F48S;R77Q	L26I;V27A;S31N	F48S;R77Q	M2 A/Duck/Guangdong/E1/2012(H10N8)
NA	T9I;G11S;V13I;M15L;T16I;I17N;M19I;N21S;I23M;L40I;I46T;N59K;T72I;A75V;A76V;G77E;S79E;V81I;S82P;K84N;N88S;S90P;K111R;P126H;L140M;I188T;G210S;A232V;R257K;I263V;M269L;N270D;S286G;T289I;E311D;I321V;F322L;K331R;V338I;S340I;I365T;E398D;D416N;I418M;G454S		N88S;S90P;I365T	NA A/California/07/2009(H1N1)
NP	A22T;G34E;K48Q;R98K;R99K;E114D;M189L;A284I;A323V;R351K;V353I;Q357K;V363I;S377I;R384K;A423T;R452K;S482N;N498S		R99K;Q357K	NP A/Duck/HongKong/24/1976(H4N2)
NS	R21Q;D24N;Q25W;E26G;G47S;S48N;D53E;T56P;E66K;R67C;S73Y;M79I;I81T;A86T;R88H;M98I;V111L;A112T;S114P;I123V;D152E;V180I;G183K;T197A;S206T;D209I;L214F;P215I;P216T;A223E;V226I;E227G;S228P		I81T;P215I;A223E;V226I;E227G;S228P	NS1 A/Shearwater/Australia/2576/1979(H15N9)
NS	K18R;E26K;I31M;V49L;M52S;L55H;V83I;L120F		K18R;M52S	NS2 A/WSN//1933(H1N1)
PA	I13V;L42M;R57K;I61T;G66S;V94I;S184L;G186A;E206D;R212H;D216N;E252G;K262R;T263E;N321H;I323V;L335I;Q346N;M374V;V387I;E399K;P400V;I432V;I438V;I505V;L549I;A553S;T618V;T639A;P653S;E688D;T712M		R57K;P400V	PA A/Herringgull/NewJersey/780/1986(H16N3)
PB1	K52R;K54R;I69V;S152M;G154S;V191I;Q210H;R211K;T257A;S375D;I423V;R486K;I517V;T566M;R584H;E618V;Q621R;V632I;S633N;V640I;V645I;A648S;I682V;I735M		S375D	PB1 A/Duck/Alberta/60/1976(H12N5)
PB2	S12L;K61R;T76I;S107D;R251K;T271I;S286G;I292M;I295V;R299K;I338V;K353R;R389K;C409R;V457I;M473V;I478V;V480I;M483T;Q507P;V584I;D611E;M645L;V649I;D701N;K718R;I756L	D701N	I292M	PB2 A/Duck/Alberta/60/1976(H12N5)
